# Flexibility in the perceptual span during reading: Evidence from Mongolian

**DOI:** 10.3758/s13414-019-01960-9

**Published:** 2020-01-02

**Authors:** Juan Su, Guoen Yin, Xuejun Bai, Guoli Yan, Stoyan Kurtev, Kayleigh L. Warrington, Victoria A. McGowan, Simon P. Liversedge, Kevin B. Paterson

**Affiliations:** 1 Academy of Psychology and Behavior, Tianjin Normal University, Tianjin, China; 2 College of Statistics and Mathematics, Inner Mongolia University of Finance and Economics, Hohhot, Inner Mongolia China; 3 Faculty Research Centre for Psychology, Behaviour and Achievement, Coventry University, Coventry, UK; 4 Department of Neuroscience, Psychology and Behaviour, University of Leicester, Leicester, UK; 5 Department of Psychology, University of Central Lancashire, Preston, UK

**Keywords:** Eye movements during reading, Perceptual span, Vertical reading, Mongolian

## Abstract

Readers can acquire useful information from only a narrow region of text around each fixation (the *perceptual span*), which extends asymmetrically in the direction of reading. Studies with bilingual readers have additionally shown that this asymmetry reverses with changes in horizontal reading direction. However, little is known about the perceptual span’s flexibility following orthogonal (vertical vs. horizontal) changes in reading direction, because of the scarcity of vertical writing systems and because changes in reading direction often are confounded with text orientation. Accordingly, we assessed effects in a language (Mongolian) that avoids this confound, in which text is conventionally read vertically but can also be read horizontally. Sentences were presented normally or in a gaze-contingent paradigm in which a restricted region of text was displayed normally around each fixation and other text was degraded. The perceptual span effects on reading rates were similar in both reading directions. These findings therefore provide a unique (nonconfounded) demonstration of perceptual span flexibility.

During reading, the eyes make high-velocity movements (saccades) separated by brief fixational pauses (for reviews, see Liversedge & Findlay, 2000; Rayner, 1998, 2009). Readers make these eye movements because they can acquire only a little useful information on each fixational pause, due to limitations in retinal acuity (see, e.g., Hilz & Cavonius, 1974), and so must make multiple fixations in order to process each line of text. The area from which this useful information can be acquired on each fixation (the *perceptual span*) has been widely studied using gaze-contingent paradigms (for a review, see Rayner, 2014). In these, text is presented normally only within a narrow region (window) around each fixation, and the letters outside this window are masked (e.g., by replacing each with an “x”), with the window size varying across an experiment. These windows are yoked to the reader’s eye movements, so that when the eyes move to fixate a new location, the text within the window at this new location is shown normally and text outside the window is masked.

Following the logic that the windows that produce normal reading rates must encompass the perceptual span, research using this paradigm has shown that skilled readers of English obtain useful information from an asymmetric area extending about 14–15 letters to the right of fixation and 3–4 letters to the left (McConkie & Rayner, 1975, 1976; Rayner, Well, & Pollatsek, 1980). Research with bilingual readers of English and of languages read from right to left (i.e., Arabic, Hebrew, Urdu) has additionally shown that this asymmetry can be reversed by changing the horizontal reading direction (Jordan et al., 2014; Paterson et al., 2014; Pollatsek, Bolozky, Well, & Rayner, 1981). Such findings demonstrate that the perceptual span can adjust flexibly to changes in reading direction, through a greater allocation of visual attention in the direction of reading to facilitate the preprocessing of upcoming text and the programming of forward-moving saccades (Rayner, Well, Pollatsek, & Bertera, 1982). However, the extent to which the perceptual span can adjust to accommodate orthogonal changes in reading direction (vertical vs. horizontal reading) has received little attention, most likely due to the scarcity of vertical writing systems.

There nevertheless are good reasons to believe that reading vertically is less efficient than reading horizontally. Nonreading studies have suggested that oculomotor control is poorer in the vertical than in the horizontal plane (Collewijn, Erkelens, & Steinman, 1988), and that asymmetries in upper- and lower-hemifield sensitivities to visual and linguistic information might impair vertical reading (e.g., Goldstein & Babkoff, 2001; Hagenbeek & Van Strien, 2002; Yu, Legge, Wagoner, & Chung, 2014). However, investigations of reading direction effects in alphabetic languages have been confounded by readers’ unfamiliarity with vertical text (Huey, 1898; Schmidt, Ullrich, & Rossner, 1993; Tinker, 1955; Yu, Park, Gerold, & Legge, 2010), and the limited evidence available to date has come from studies with character-based languages, such as Japanese, that commonly use both horizontal and vertical text formats (Osaka & Oda, 1991). This research in Japanese has reported similar perceptual span effects in horizontal and vertical reading. However, the conclusions that can be drawn are limited, because of the comparatively small perceptual span (five or fewer characters in Japanese) and, more importantly, because characters maintain an upright orientation in both the horizontal and vertical formats, so that reading direction is confounded with character orientation in this language.

Accordingly, to avoid the confounds present in all previous studies, it would be necessary to employ a language that uses both horizontal and vertical text in which the orientation of the orthographic units (i.e., letters, words) is always consistent with the reading direction. One language with this rare characteristic is Mongolian, which uses an alphabetic writing system derived from Old Uyghur, printed using a proportional semicursive script in which letters are connected by ligatures (i.e., short strokes; see Campbell, 1997). Crucially, Mongolian conventionally is printed vertically and read from top to bottom, but it can be printed and read horizontally (from left to right). Horizontal text presentations effectively are created by rotating the vertical text counterclockwise through 90°, thereby maintaining consistency of reading direction with the orientation of orthography, so that Mongolian is ideally suited to studying perceptual span effects during vertical and horizontal alphabetic reading. We therefore examined reading direction effects on the perceptual span for this language, using skilled native Mongolian readers and a gaze-contingent moving-window paradigm in which sentences were presented vertically or horizontally, either entirely as normal or normal only within a narrow window around fixation, with text outside the window visually degraded. Following previous research (Jordan et al., 2014; Paterson et al., 2014), window size was varied systematically across the experiment, to enable comparisons between a baseline symmetrical window and windows extending asymmetrically in the same or in the opposite direction from that of reading. We predicted faster reading rates for the more familiar vertical displays. We also predicted that reading rates would be closest to normal for windows extending asymmetrically in the direction of reading, and slower for both baseline symmetrical windows and windows extending asymmetrically opposite to the direction of reading. Moreover, such effects should be similar for horizontal and vertical reading if the perceptual span adapts flexibly to this orthogonal change in reading direction.

## Method

### Ethics statement

The study received ethical approval from the research ethics committee of the School of Psychology at the University of Leicester (UK) and was conducted in accordance with the principles of the Declaration of Helsinki.

### Participants

The participants were 24 native Mongolian speakers (18–21 years of age) from Inner Mongolia University of Finance and Economics in Hohhot, Inner Mongolia, China. These participants averaged 15 years (*SD =* 2) experience with reading Mongolian and had normal or corrected-to-normal visual acuity. Participants reported 85% (*SD =* 10%) daily exposure to Mongolian text and 15% (*SD =* 8%) exposure to other scripts (mostly Chinese and English). The sample size comfortably exceeded the sample sizes in previous perceptual span studies (6 in McConkie & Rayner, 1975; 3 in McConkie & Rayner, 1976; 6 in Pollatsek et al., 1981; 7, 9, and 14 in each of three experiments by Rayner et al., 1980; and 12 each in Jordan et al., 2014, and Paterson et al., 2014). No previous studies have investigated perceptual span effects in Mongolian reading, so we performed a power analysis using the SIMR package (Green & Macleod, 2016) in R (R Core Team, 2016) and Arabic data from a study with Arabic–English bilinguals reported by Jordan et al. (2014). We computed the sample size required to detect slower reading speeds, relative to normal text displays, for a baseline symmetrical window and for asymmetric windows extending counter to the direction of reading, as these were comparable to the conditions expected to produce slower reading speeds in the present experiment. The results indicated that the Jordan et al. experiment was well-powered (power > 80%) and that a sample size of three participants in a within-participants design would be sufficient to detect effects of similar size in the present experiment.

### Stimuli and design

The stimuli were 120 Mongolian sentences. These were 10–14 words long (*M =* 11), presented as a single line of text in a commonly used 20-point fixed-width font (Mongolian Baiti). Horizontal sentences were displayed to be read from left to right, and vertical sentences were displayed to be read from top to bottom. The sentences were presented either entirely as normal or using a gaze-contingent moving window paradigm in which the text was presented as normal only within a narrow region (window) around fixation. Five windows were used: a small symmetrical window; two asymmetric windows extending leftward or upward, depending on the text orientation; and two asymmetric windows extending rightward or downward, depending on the text orientation. The window sizes were the same as in the studies of perceptual span effects in Arabic and Urdu by Jordan et al. (2014) and Paterson et al. (2014). The symmetrical window provided a baseline small-window condition and extended 0.5° on each side of fixation (0.5_0.5 window). The leftward or upward asymmetric windows (i.e., elongated counter to the direction of reading) extended either 1.5° to the left/up and 0.5° to the right/down (1.5_0.5 windows) or 2.5° to the left/up and 0.5° to the right/down (2.5_0.5 windows). The rightward or downward asymmetric windows (i.e., elongated in the direction of reading) extended either 0.5° to the left/up and 1.5° to the right/down (0.5_1.5 windows) or 0.5° to the left/up and 2.5° to the right/down (0.5_2.5 windows). The text outside each window was blurred (using MATLAB), so that the location and shape of the words was preserved but letter identities were obscured (for more information, see Jordan et al., 2014; Paterson et al., 2014). Ten Mongolian readers who did not take part in the experiment reported being unable to read similarly blurred text. At the viewing distance in the experiment, 2.5° encompassed approximately ten letter spaces. Custom software ensured that each window moved in close synchrony with each reader’s eye movements and that display changes were made rapidly (within 10–12 ms). The phenomenological experience for all participants was that the windows moved in perfect synchrony with their eyes.

**Fig. 1:**
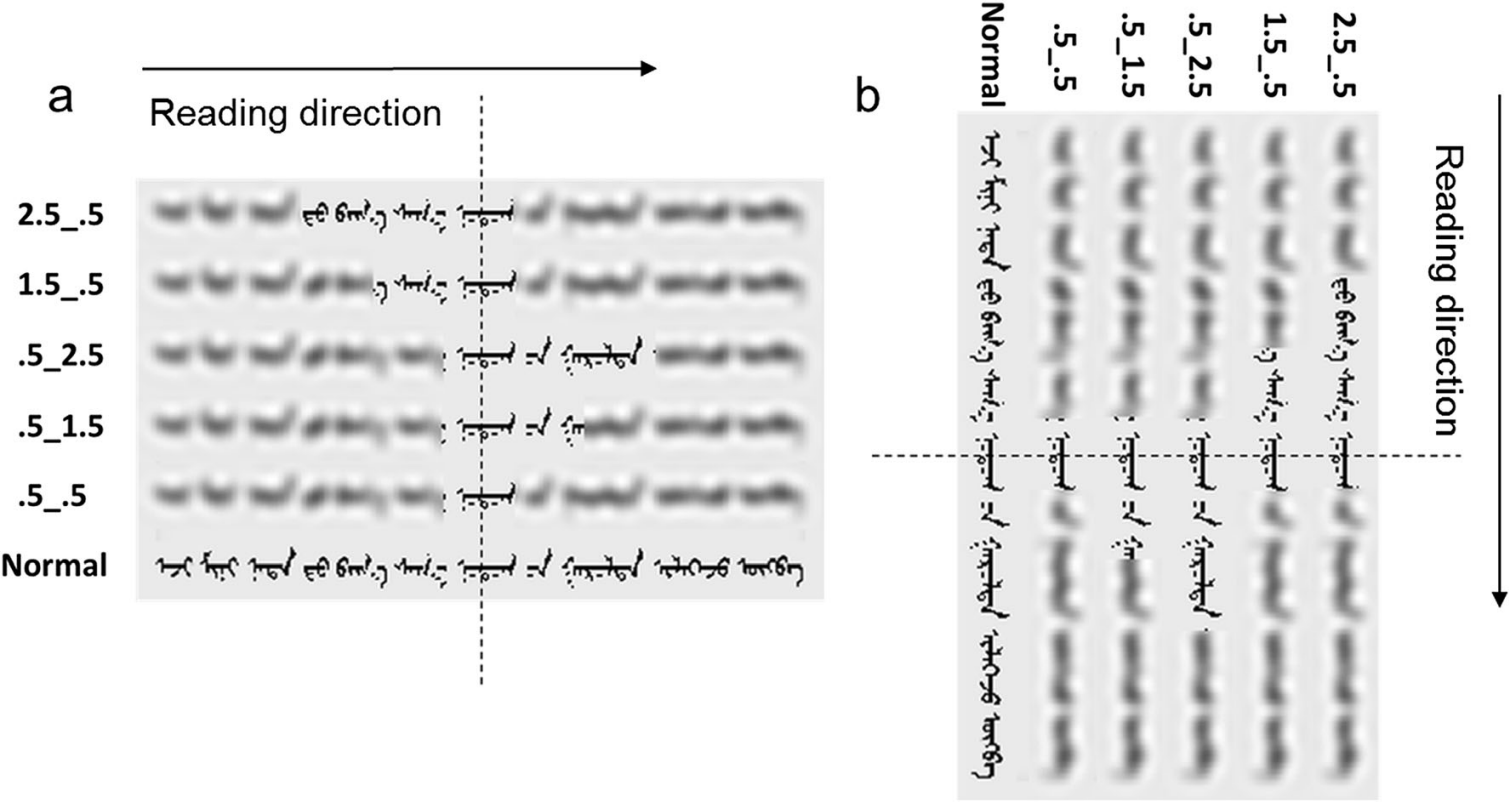
Examples of sentences in each display condition, presented in the (a) horizontal and (b) vertical text orientations. A reader’s fixation location is denoted by a dashed line. The text within each moving window around this point is shown normally, and the text outside the window is blurred. The example sentence translates into English as “My mother sent a few special local products to me.”

The sentence stimuli were randomized and sampled using a Latin square design so that each participant saw half the sentences in a vertical orientation and half in a horizontal orientation, and ten of the sentences in each orientation were shown in each display condition. This ensured that each sentence was shown only once to each participant, and that all sentences were seen equal numbers of times in vertical and horizontal displays across the experiment. Sentences in each orientation were presented in separate sessions in a randomized order, and the order of sessions was counterbalanced across participants. An additional 12 sentences were used as practice items at the beginning of each session. The experiment therefore had a within-participants design with the factors text orientation (vertical, horizontal) and display condition (normal, 0.5_0.5 window, 1.5_0.5 window, 2.5_0.5 window, 0.5_1.5 window, 0.5_2.5 window) (see Fig. 1).

### Apparatus and procedure

An EyeLink 1000 eyetracker recorded the right-eye gaze location every millisecond during binocular viewing. Sentences were displayed on a high-performance ViewSonic monitor (1,024 × 768 resolution) with a fast vertical refresh rate (120 Hz). Before the start of the experiment, each participant sat in front of the display screen and was instructed to read normally and for comprehension. At the start of each session, the eyetracker was calibrated to the participant’s eye movements, using a horizontal or vertical five-point calibration procedure as appropriate, and calibration accuracy was checked before each trial. If necessary, the eyetracker was then recalibrated to ensure a high degree of spatial accuracy (< 0.30° error) across the experiment.

At the start of each trial, a fixation square equal in size to one letter space was presented near the top of the screen (for vertical presentations) or the left side of the screen (for horizontal presentations). Once this was fixated, a sentence was displayed with the first letter replacing the fixation square. The participant pressed a response key after finishing reading each sentence, and the sentence disappeared. On 30% of trials, the sentence was replaced by a comprehension question, to which participants responded by pressing a “yes” or “no” response key. Participants received short breaks between sessions. For each participant, the experiment lasted approximately 40 min.

## Results

**Fig. 2:**
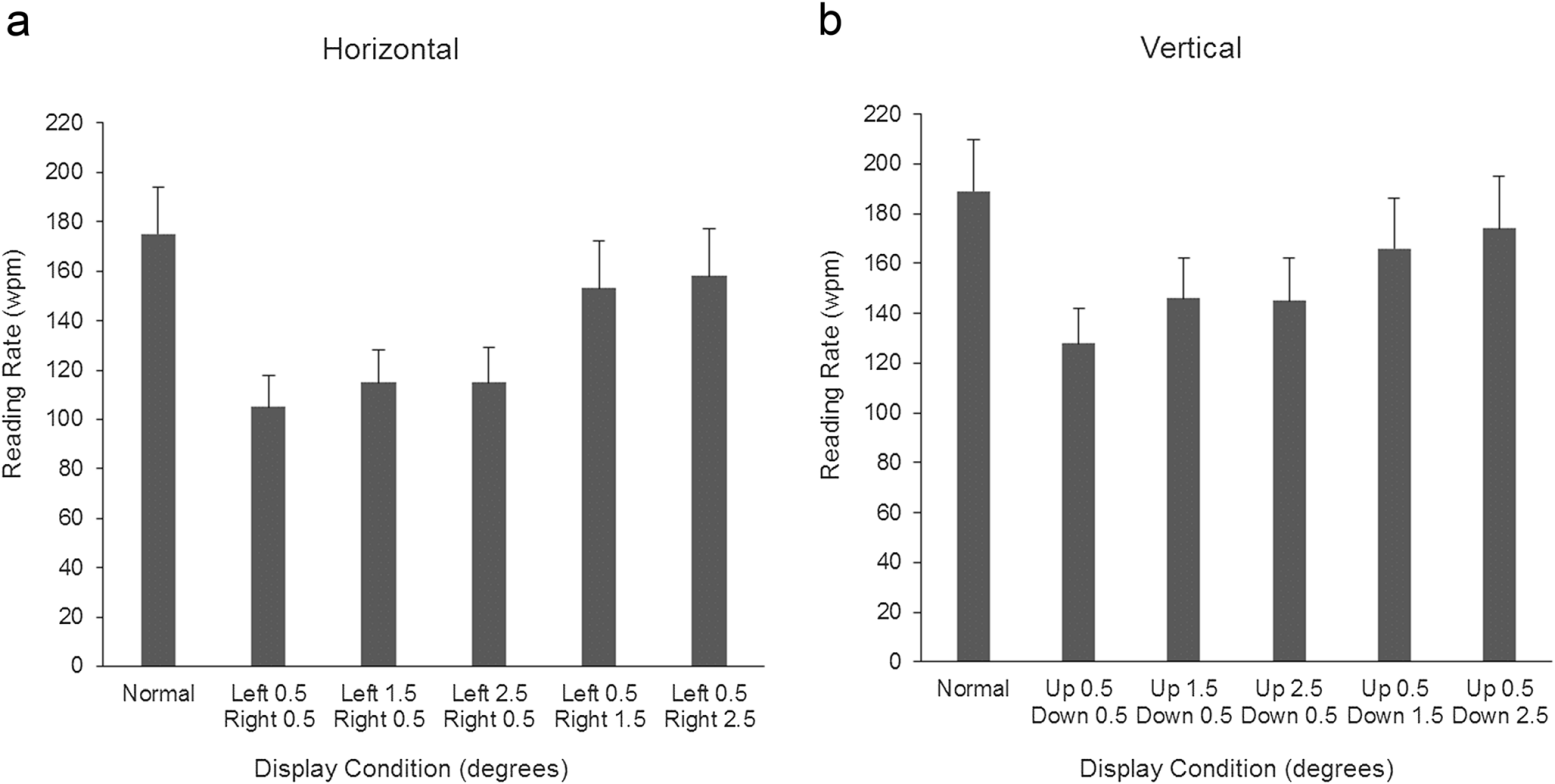
Mean reading rates (in words per minute, wpm) for each display condition in the (a) horizontal and (b) vertical text orientations. Error bars represent 95% confidence intervals. Means and confidence intervals were calculated using estimates generated by the lsmeans package (version 1.3.5.1; Lenth, 2016) in R

Participants showed similarly high levels of response accuracy for comprehension questions that followed the sentences in each text orientation (horizontal = 87% correct, vertical = 91% correct). Reading rate (words per minute) provides the most comprehensive and informative measure of reading performance in gaze-contingent moving-window experiments (see McConkie & Rayner, 1975), so these are reported. Reading rates for text in each display condition in the horizontal and vertical orientations are shown in Fig. 2.

These were analyzed by constructing linear mixed-effects models (LMMs; Baayen, Davidson, & Bates, 2008) using the lme4 package (Bates, Mächler, Bolker, & Walker, 2014) in R (R version 3.6.1, R Studio version 1.2.1335; R Core Team, 2016). The analyses included participants and stimuli as random effects, and text orientation and display condition as fixed effects. A model was coded to assess the main effect of text orientation, using a contrast matrix to assess differential effects of display condition and their interactions with text orientation. The contrast matrix compared display conditions in turn, first by comparing the display condition with the slowest reading rate (0.5_0.5 windows) against the one with the next fastest reading rate (1.5_0.5 windows), and then progressively comparing each slower condition with the next fastest one (resulting in comparisons of 0.5_0.5 vs. 1.5_0.5, 1.5_0.5 vs. 2.5_0.5, 2.5_0.5 vs. 0.5_1.5, 0.5_1.5 vs. 0.5_2.5, and 0.5_2.5 windows vs. the normal text display). A second contrast matrix examined the interaction between each of these comparisons and text orientation. Wherever any interaction was observed, the effects for the relevant display conditions were examined separately for each text orientation. The reading rate data were log-transformed, but analyses of the transformed and untransformed data produced the same pattern of effects, so for transparency, only effects for the untransformed data are presented. A maximal random-effects model did not converge, so effects are reported for a random-effects model that included a random slope for text orientation but no random slopes for display condition (Barr, Levy, Scheepers, & Tily, 2013).

A main effect of text orientation (*β* = 20.78, 95% confidence interval [CI] = [11.20, 30.37], *SE* = 4.78, *t* = 4.34) was due to faster reading rates for vertical than for horizontal text displays, most likely because the native Mongolian readers had greater familiarity with the more conventional vertical text format. For the main effect of display condition, small symmetrical windows (0.5_0.5 windows) produced the slowest reading rates. Compared to the small symmetrical windows, reading rates were faster for small leftward or upward asymmetric windows (0.5_0.5 vs. 1.5_0.5 windows, *β* = 13.70, 95% CI = [8.75, 18.66], *SE* = 2.53, *t* = 5.41), although there were no increased benefit for the large leftward or upward asymmetric windows (1.5_0.5 vs. 2.5_0.5 windows; *β* = – 0.13, 95% CI = [– 5.09, 4.82], *SE* = 2.53, *t* = 0.05). As compared to the large leftward or upward asymmetric windows, small rightward or downward asymmetric windows produced faster reading rates (i.e., 2.5_0.5 vs. 0.5_1.5 windows, *β* = 29.27, 95% CI = [24.31, 34.23], *SE* = 2.53, *t* = 11.55), while large rightward or downward asymmetric windows produced even faster reading rates (0.5_1.5 vs. 0.5_2.5 windows, *β* = 6.60, 95% CI = [1.65, 11.54], *SE* = 2.53, *t* = 2.61). However, even these windows did not produce reading rates as fast as text presented normally (i.e., normal vs. 0.5_2.5 windows, *β* = 16.49, CI = [11.55, 21.43], *SE* = 2.52, *t* = 6.53). Accordingly, windows that extended asymmetrically farther in the direction of reading (i.e., downward for vertical displays, rightward for horizontal displays) produced the fastest reading rates in the moving-window paradigm, albeit still a little slower than when text was presented entirely as normal. The findings therefore clearly show that the perceptual span can adjust flexibility to accommodate orthogonal changes in reading direction in vertical as compared to horizontal reading.

Crucially, the pattern of effects we observed was essentially the same across horizontal and vertical presentations, so there was little indication that perceptual span effects differed as a function of reading direction. Indeed, only one interaction effect was observed, between text orientation and a comparison of large leftward/upward asymmetric windows and small rightward/downward asymmetric windows (i.e., text orientation × 2.5_0.5 vs. 0.5_1.5 windows; *β* = – 17.78, 95% CI = [– 27.69, – 7.86], *SE* = 5.07, *t* = 3.51). This interaction was due to a larger reading rate advantage for the small rightward/downward asymmetric windows than for the large leftward/upward asymmetric windows in horizontal as compared to vertical displays, although an advantage for rightward/downward asymmetric windows was observed in both text orientations (vertical, *β* = 38.13, *SE* = 3.72, *t* = 10.24; horizontal, *β* = 20.44, *SE* = 3.71, *t* = 5.51). Figure 2 suggests that readers had generally faster reading rates for windows extending asymmetrically leftward/upward in vertical as compared to horizontal reading directions. They therefore may have been less disrupted by windows that extended counter to the reading direction in more familiar vertical text formats. No other interactions with text orientation were significant (all *β*s < 8, *SE*s < 5.1, *t*s > 1.4).

In addition to the LMM analyses, Bayes factors (Kass & Raftery, 1995) were computed in order to assess the strength of evidence for models that included an interaction with text orientation against alternative models without this interaction effect. These were performed using the BayesFactor package (version 0.9.12-2; Rouder, Morey, Speckman, & Province, 2012) in R. Marginal likelihood was obtained using Monte Carlo sampling, with iterations set at 100,000 and the scaling factor for *g*-priors set to 0.5. Participants and stimuli were specified as random variables. Model comparisons were made using standard interpretation categories (Vandekerckhove, Matzke, & Wagenmakers, 2014; derived from Jeffreys, 1961), such that Bayes factors (BFs) > 3 were taken to provide weak to moderate support for a model over an alternative model, and BFs > 10 to provide strong support, whereas BFs < 1 were taken to provide evidence against a model and in favor of the alternative model. The results were in line with the LMM analyses and provided strong support for a model that included an interaction effect for 2.5_0.5 windows versus 0.5_1.5 windows (BF = 30.2). As we explained above, this interaction was not of theoretical interest. Crucially, all other comparisons favored a model with main effects but no interaction (all BFs < 0.5), indicating that the reading speed advantages for windows extending asymmetrically in the direction of reading were similar for both horizontal and vertical text displays.

## Discussion

A small number of previous studies have shown that the perceptual span can adjust flexibly to changes in horizontal reading direction for bilingual readers of English and right-to-left languages (i.e., Arabic, Hebrew, and Urdu; Jordan et al., 2014; Paterson et al., 2014; Pollatsek et al., 1981). The present experiment extended these findings by demonstrating that the perceptual span can also adjust flexibly to accommodate orthogonal change in vertical as compared to horizontal reading directions in a language (Mongolian) in which the reading direction is always consistent with the orthographic orientation and in which both vertical and horizontal text formats are commonly used. In particular, although Mongolian was read faster in vertical than in horizontal orientations, most likely due to the former orientation’s greater familiarity, reading rates were closest to normal for both vertical and horizontal reading directions when text was presented normally within a moving-window display that extended asymmetrically in the direction of reading and in which text outside this window was visually degraded. Moreover, the perceptual span effects were essentially the same in both reading directions. The experiment therefore provides a unique demonstration of the flexibility with which perceptual mechanisms that underlie reading can adjust flexibly to accommodate changes in reading direction.

The findings effectively replicate those reported by Osaka and Oda (1991), showing similarities between vertical and horizontal perceptual spans when reading Japanese, only in an alphabetic script that maintains consistency of reading direction and orthographic orientation. Future research to disentangle the effects of orthographic orientation would be difficult to conduct with a semicursive script like Mongolian, as changing the normal orientation of letters would also disrupt the integrity of words. This might be possible, however, by varying reading direction and character orientation orthogonally in a nonalphabetic language (e.g., Chinese, Japanese) that can be read vertically or horizontally, although how easily characters in these languages could be recognized when displayed in unfamiliar orientations is unclear (see Zhang, Yuan, Bao, & Zhang, 2008).
